# Adult Onset Vanishing White Matter Disease: A Rare Case Report

**DOI:** 10.7759/cureus.30177

**Published:** 2022-10-11

**Authors:** Govind Nagdev, Rajeshwari S Vhora, Gajanan Chavan, Gaurav Sahu

**Affiliations:** 1 Department of Emergency Medicine, Jawaharlal Nehru Medical College, Datta Meghe Institute of Medical Sciences, Wardha, IND; 2 Department of Emergency Medicine, B. J. Medical College & Sassoon General Hospital, Pune, IND; 3 Department of Medicine, Jawaharlal Nehru Medical College, Datta Meghe Institute of Medical Sciences, Wardha, IND

**Keywords:** india, psychiatric, progressive ataxia, cerebral leukodystrophy, vanishing white matter

## Abstract

Vanishing white matter disease (VWMD) is the most common childhood-onset inheritable progressive leukodystrophy disorder, which exclusively affects the white matter of the brain. It shows mutations in one of the five eukaryotic translation initiation factor *2B1-5 genes* following an autosomal recessive pattern, of which eIF2B5 mutation is the most frequent. These genes play a vital role in the translation and regulation of protein synthesis and mutation in them leads to a dysregulation of the cellular stress response, which in particular disrupts myelination and affects oligodendrocytes and astrocytes while sparing the neurons. Stressful situations, for example, head trauma, sudden fright, acute psychological stress, or infection, provoke severe and rapid neurological deterioration. Although it is more common in childhood, we report a case of an adult presenting with signs and symptoms of VWMD, such as abusive behavior, emotional liability, and motor incoordination. To our knowledge, this is the first case of adult-onset VWMD in Maharashtra, India, confirmed by magnetic resonance imaging (MRI) of the brain.

## Introduction

Vanishing white matter disease (VWMD) is a progressive leukodystrophy exclusively affecting the white matter of the brain, mostly reported in children as a congenital form or early to late childhood-onset type incidence ranging from 1.2 to 3.01 per 100,000 persons per year [1]. Only China and Japan have documented incidence in Asia [2]. There have only been a few cases documented in India so far [3-5]. Adult-onset VWM is thought to account for 15% of cases globally [6], and so far, only two adults, both presented by Shivaram S et al., have been reported in India [7]. VWMD is caused by a mutation in one of the five eukaryotic translation initiation factor 2B genes following an autosomal recessive pattern, of which eIF2B5 mutations are the most frequent [8]. Along with GTP and methionyl-transfer RNA (MettRNAi) [9], eIF2 forms a ternary complex which is responsible for the initiation of translation of mRNAs into polypeptides [10]. Under various stress situations, the translation initiation point proves to be a crucial step in the regulation of protein synthesis [11]. The cellular-stress response, a defence mechanism for cells, which involves the inhibition of protein synthesis and thereby enhances cell survival by limiting the accumulation of denatured proteins and saving cellular energy. Mutation of eIF2B genes leads to dysregulation of this mechanism, which in particular disrupts myelination and affects oligodendrocytes and astrocytes while sparing neurons [12]. Stressful situations such as head trauma, sudden fright, acute psychological stress or infection provoke severe and rapid neurological deterioration [13-15]. The course of the disease is variable, and the age group affected ranges from infantile to juvenile age group [16]. Although the latest reported beginning of VWMD in adults is 55 years old, it is becoming more widely recognized in adults, manifesting large phenotypic variability [17]. VWMD is also known as childhood ataxia with central hypomyelination (CACH) [12], as the most common age group involved is 2 to 6 years of age and classically presents with progressive cerebellar ataxia, epilepsy and mild intellectual disability [18]. However, extensive systemic involvement can result in a number of complications, including encephalopathy, cataracts, pancreatitis, hepatosplenomegaly, renal dysplasia, and stunted growth can occur in severe forms of disease and early onset [19]. Adult onset VWMD presents with seizures, complicated migraines, cerebellar ataxia, spasticity, dementia, psychosis and mood disturbances [20]. In light of its rarity, we report a case of MRI-confirmed adult-onset VWMD with significant phenotypic variability.

## Case presentation

We report a case of a 40-year-old woman, a resident of Amravati district, Maharashtra, India, who was brought to our hospital with a history of progressive ataxia for two years, recurrent falls, and postural instability. She used to drag her feet while walking. She was diagnosed with major depression disorder five years back, since then, she is on anti-depressants medications (Selective serotonin reuptake inhibitors [SSRIs]) for the same. There was no significant family history of psychiatric or neurological disorders. On enquiring further, her relatives mentioned her depressed mood, irritability, and uncontrollable anger. Adding to this, they indicated that she had a shorter attention span and a lower level of organizational capacity.

She was born after an uneventful normal pregnancy. She did not have any abnormalities during the neonatal period and was fully immunized according to the National Immunization schedule (NIS).

**Figure 1:**
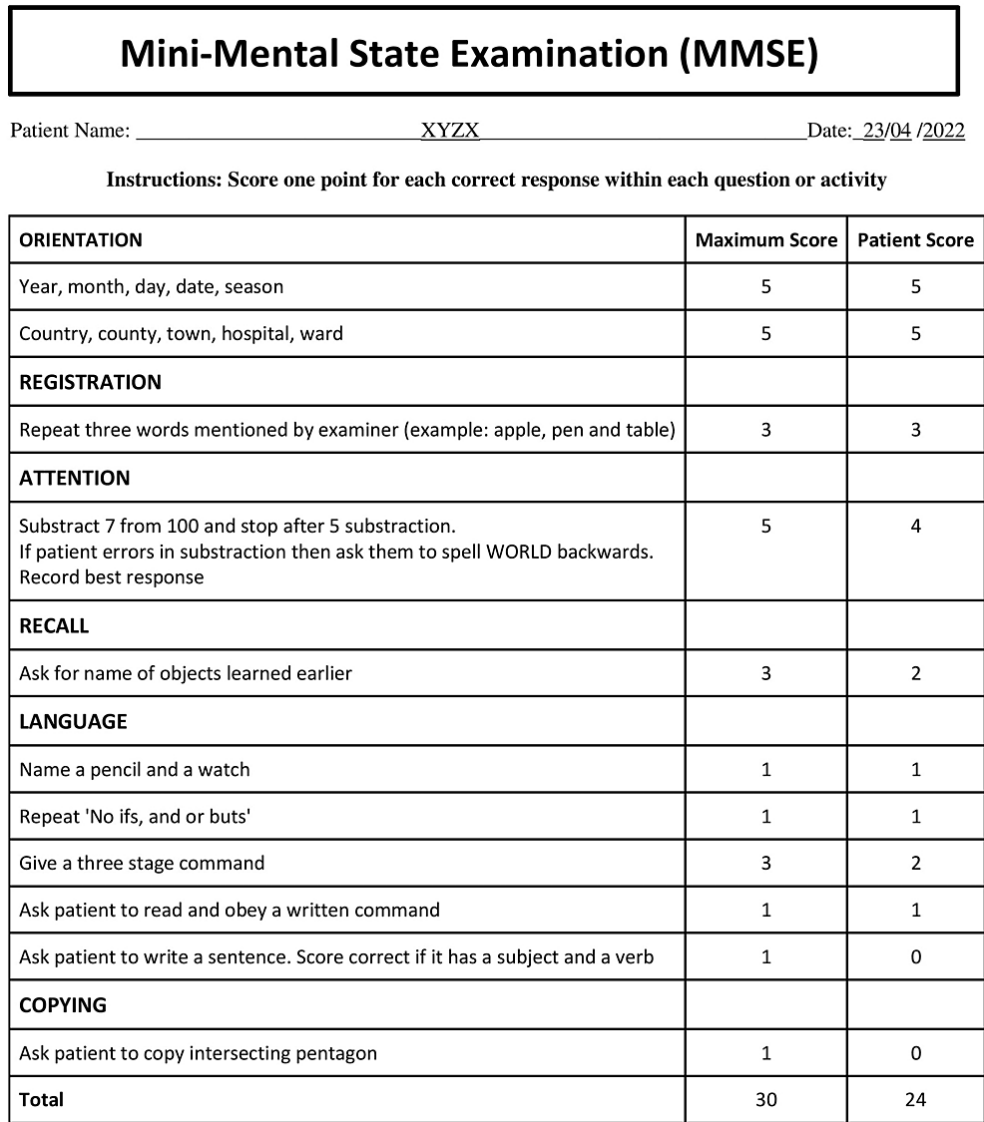
Mini-mental state examination (MMSE) questionnaire shows MMSE test, a 30-point questionnaire that was used for grading the cognitive state of patient.

On taking obstetrics and gynecological history, she was nulliparous and had an abnormal menstrual history with irregular cycles. Her general examinations were normal. Central nervous system (CNS) examination showed dystonia in the lower limbs associated with dystonic gait. Her reflexes were exaggerated. On MMSE (Mini-Mental State Examination), her score was 24/30. There was marked impairment in executive function, working memory, and processing speed (Figure 1).

**Figure 2:**
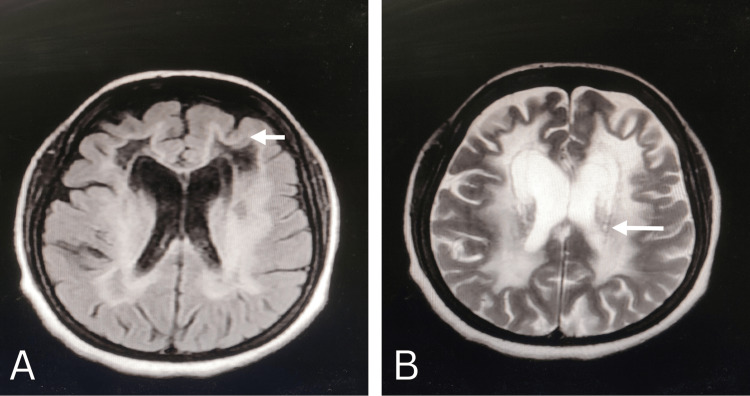
Magnetic resonance imaging findings in classic vanishing white matter (VWM) disease. (A): Axial T1-weighted image showing the diffuse abnormality (hypointense) of the white matter without involving grey matter (B): Axial T2-weighted image shows the hyperintense white matter projecting similar signals like cerebrospinal fluid.

**Figure 3:**
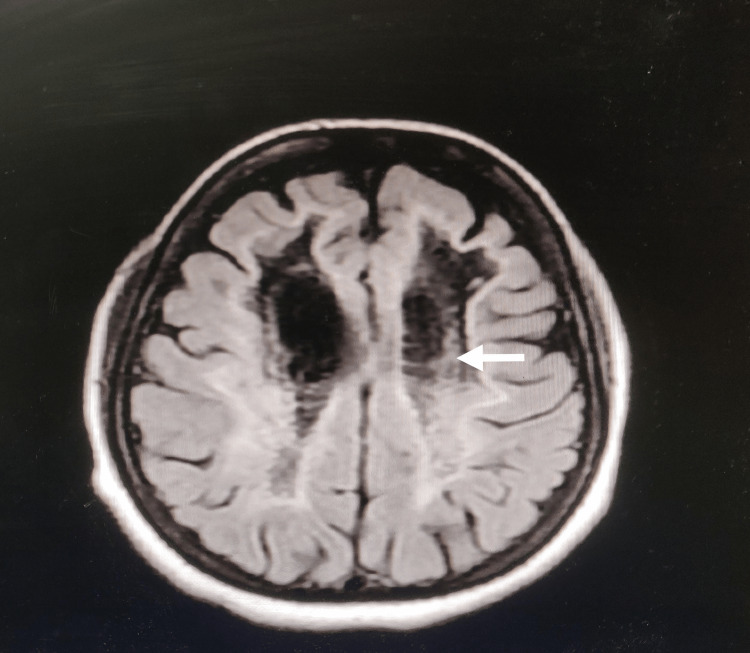
Axial FLAIR image showing cystic degeneration and rarefaction of white matter. FLAIR: Fluid-attenuated inversion recovery

Her routine investigations, including hormonal assays, were normal. MRI-brain suggested diffuse abnormality of white matter (Figure 2A, 2B) and cystic degeneration along with rarefaction of white matter (Figure 3). The grey matter was not affected. She was diagnosed with VWMD on the basis of classic findings in history, clinical examination, radiographic imaging and genetic testing with EIF2B5 gene mutation. She was discharged on SSRI (Selective Serotonin Reuptake Inhibitor) Sertraline after a neuropsychiatric consultation since no definitive treatment is established till now.

## Discussion

This case depicts vague presentation and wide phenotypic variation in adult-onset VWMD. The disease is prevalent in childhood, but adult-onset VWMD is increasingly recognized with high severity [21]. Despite little literature available on studies, the longitudinal investigations on the natural history of VWMD have shown that one of the most important prognostic factors is the age at onset [22]. Children with early-onset of the disease have rapid progressing motor dysfunction in contrast to late-onset VWMD [23]. On the contrary, in other leukodystrophies, the higher the age of onset of symptoms less severe the disease and its progression [24]. The slow disease progression in our patient was probably attributed to her late onset of symptoms and absence of any stress-provoking factor, which is consistent with other leukodystrophies. As depicted above, adult-onset VWMD presents more prominent psychiatric and cognitive symptoms as compared to early-onset disease with predominant motor disability [25]. Abnormal menstruation can precede neurological symptoms, and it is reported in females with VWMD attributed to ovarian dysfunction. This is termed ovarioleukodystrophy and is extremely uncommon, as less than 30 cases have been reported till now [26]. This patient had long-term menometrorrhagia before the onset of neurological as well as psychiatric symptoms for which ultrasonography (USG) abdomen pelvis/computed tomography (CT) abdomen-pelvis was not done.

Due to the peculiar and typical imaging patterns, which are extremely sensitive and specific for the disease, MRI has proved to be a crucial tool in diagnosing VWMD. This can be extremely useful when access to genetic testing is limited, and there aren't many other reliable biomarkers available, which can be difficult to find (like asialotransferrin and glycine, which are obtained only via the cerebrospinal fluid (CSF). Typical MRI findings are diffuse hypointense on T1-weighted imaging, hyperintense on T2-weighted imaging, without contrast enhancement along with the presence of a cavitary appearance on the FLAIR and producing CSF-like signals in all sequences is suggestive of white matter rarefaction and cystic degeneration [18,27,28]. These findings were consistent with our findings. These findings need to be differentiated from other types of leukodystrophies, like mitochondrial leukodystrophies, that on the contrary, have more restricted diffusion and show focal contrast enhancement [29]. Other differentials can be multiple system atrophy-cerebellar variants (MSA-C), sporadic adult-onset ataxia of unknown etiology (SAOA), alcoholic cerebellar degeneration (ACD), and acute post-infectious cerebellar ataxia (APCA).

Due to this wide phenotypic variation in its presentation seen in adult-onset VWMD, it can be challenging to diagnose it clinically, and therefore psychiatrists, physicians, and gynecologists should be aware of the existence of VWMD.

## Conclusions

In conclusion, VWMD is a rare neurodegenerative disease and can be diagnosed primarily based on the clinical and pathognomic patterns on MRI. A comprehensive neurological examination is warranted in every patient with the above presentation, as the first point of contact for such patients will be a psychiatrist. For a patient with cognitive impairments, mood disturbances, and motor disorders, VWMD should be kept as a differential diagnosis. Early detection can possibly facilitate not curative but preventive measures as the disease can rapidly progress with stress factors such as emotional disturbances, head injury, seizures, etc.
